# Current trends in diagnostic and therapeutic management of the axilla in breast cancer patients receiving neoadjuvant therapy: results of the German-wide NOGGO MONITOR 24 survey

**DOI:** 10.1007/s00404-022-06804-w

**Published:** 2022-10-10

**Authors:** Maggie Banys-Paluchowski, Michael Untch, Natalia Krawczyk, Maria Thurmann, Thorsten Kühn, Jalid Sehouli, Maria Luisa Gasparri, Jana de Boniface, Oreste Davide Gentilini, Elmar Stickeler, Nina Ditsch, Achim Rody, Peter Paluchowski, Jens-Uwe Blohmer

**Affiliations:** 1 Department of Gynecology and Obstetrics, University Hospital Schleswig-Holstein Campus Lübeck, Ratzeburger Allee 160, 23538 Lübeck, Germany; 2 Department of Gynecology and Obstetrics, Helios Klinikum Berlin-Buch, Berlin, Germany; 3 Department of Gynecology and Obstetrics, Heinrich Heine University Düsseldorf, Düsseldorf, Germany; 4 Department of Gynecology and Obstetrics, Klinikum Esslingen, Esslingen, Germany; 5 Department of Gynecology With Center for Oncological Surgery, Corporate Member of Freie Universität Berlin, Humboldt-Universität Zu Berlin, and Berlin Institute of Health, Virchow Campus Clinic, Charité Medical University, Berlin, Germany; 6 Humboldt-Universität Zu Berlin, and Berlin Institute of Health, Freie Universität Berlin, Berlin, Germany; 7 Department of Gynecology and Obstetrics, Ente Ospedaliero Cantonale, Ospedale Regionale Di Lugano, Lugano, Switzerland; 8 Faculty of Biomedicine, University of the Italian Switzerland (USI), Lugano, Switzerland; 9 Department of Molecular Medicine and Surgery, Karolinska Institutet, Stockholm, Sweden; 10 Department of Surgery, Capio St. Göran’s Hospital, Stockholm, Sweden; 11 Breast Surgery Unit, San Raffaele Hospital Milan, Milan, Italy; 12 Department of Gynecology and Obstetrics, University Hospital Aachen, Aachen, Germany; 13 Department of Gynecology and Obstetrics, University of Augsburg, Augsburg, Germany; 14 Department of Gynecology and Obstetrics and Breast Cancer Center, Regio Klinikum Pinneberg, Pinneberg, Germany; 15 Department of Gynecology and Breast Cancer Center, Charité Universitätsmedizin Berlin, Berlin, Germany

**Keywords:** Breast cancer, Neoadjuvant chemotherapy, Targeted axillary dissection, Breast ultrasound, Survey

## Abstract

**Purpose:**

In the last 2 decades, the optimal management of the axilla in breast cancer patients receiving neoadjuvant chemotherapy (NACT) has been one of the most frequently discussed topics. Little is known about the attitudes of surgeons/radiologists towards new developments such as targeted axillary dissection. Therefore, the NOGGO conducted a survey to evaluate the current approach to axillary management.

**Methods:**

A standardized digital questionnaire was sent out to > 200 departments in Germany between 7/2021 and 5/2022. The survey was supported by EUBREAST.

**Results:**

In total, 116 physicians completed the survey. In cN0 patients scheduled to receive NACT, 89% of respondents recommended sentinel lymph node biopsy (SLNB) after NACT. In case of ypN1mi(sn), 44% advised no further therapy, while 31% proposed ALND and 25% axillary irradiation. 64% of respondents recommended a minimally invasive axillary biopsy to cN + patients. TAD was used at the departments of 82% of respondents and was offered to all cN + patients converting to ycN0 by 57% and only to selected patients, usually based on the number of suspicious nodes at time of presentation, by 43%. The most common marking technique was a clip/coil. 67% estimated that the detection rate of their marker was very good or good.

**Conclusion:**

This survey shows a heterogenous approach towards axillary management in the neoadjuvant setting in Germany. Most respondents follow current guidelines. Since only two-thirds of respondents experienced the detection rate of the marker used at their department as (very) good, future studies should focus on the comparative evaluation of different marking techniques.

**Supplementary Information:**

The online version contains supplementary material available at 10.1007/s00404-022-06804-w.

## What does this study add to the clinical work

**Table:** 

Current guidelines on axillary management in breast cancer treated with neoadjuvant chemotherapy are rapidly changing. This study is the first to examine current trends in the clinical practice in Germany.	

## Introduction

In the last 2 decades, the optimal management of the axilla in breast cancer patients has been one of the most frequently discussed topics in the surgical community. For patients receiving primary surgical therapy, sentinel lymph node biopsy (SLNB) has long replaced axillary lymph node dissection (ALND) in clinically node-negative (cN0) patients, and omission of ALND in those with 1–2 positive sentinel nodes receiving breast-conserving surgery is standard of care since the results of the ACOSOG Z0011 trial were published [1–4].

Following de-escalation of surgical treatment in the primary surgery setting, attempts have been made to define optimal management of patients receiving neoadjuvant chemotherapy (NACT) [5]. In this context, several open issues remain to be clarified. While all current guidelines recommend assessing the axilla prior to the start of NACT using both clinical examination and imaging, international recommendations differ with regard to the necessity for a cytological and/or histological confirmation of nodal involvement [1, 2, 6]. In addition, initially node-positive patients converting to clinically negative node status (cN +  → ycN0) are currently offered different surgical techniques. While some guidelines still recommend a conventional ALND in this setting, others endorse surgical de-escalation using either SLNB alone or a combination of SLNB and removal of a target lymph node marked before NACT, a strategy usually referred to as targeted axillary dissection (TAD) [5, 7]. The optimal marking technique and long-term oncological safety of TAD are, however, a matter of debate. Little is known about the attitudes of surgeons and radiologists towards these new developments. Therefore, the North-Eastern German Society of Gynecological Oncology (NOGGO, www.noggo.de) conducted a nationwide digital survey to evaluate current approach to axillary diagnostics and treatment in Germany. The survey was supported by the EUBREAST Study Group (www.eubreast.com).

## Materials and methods

Between July 2021 and May 2022, the North-Eastern Society of Gynecological Oncology carried out a nationwide online survey among gynecological and breast departments in Germany. Target groups were surgeons and radiologists involved in breast cancer diagnostics and treatment. Respondents remained anonymous. The language of the questionnaire was German. A standardized digital questionnaire consisting of 31 questions (Supplementary Table 1) was constructed using SurveyMonkey and sent out to over 200 gynecological and breast departments in Germany via e-mail. The NOGGO, as one of the largest gynecooncological study groups in Germany, has broad experience in conducting digital surveys targeting patients and/or physicians. All gynecologic and breast cancer centers which have participated in such surveys in the past and who were registered with the NOGGO were contacted to complete the survey. The invitation to complete the survey was also sent to all members of 148 German study sites participating in the international AXSANA EUBREAST-3 study (http://axsana.eubreast.com). The survey was closed on May 13th, 2022. The questionnaire was divided into three main sections: (1) baseline sociodemographic questions, (2) questions on TAD, and (3) questions on marking techniques. Two additional questions focused on clip/coil marking, and two questions focused on probe-guided localization techniques, such as magnetic, radar reflecting and radiofrequency markers. Free text answers were possible in some questions. The study was designed using advanced branching, so that some questions were not shown depending on the answers to previous ones. The survey was approved by the Charité Ethics Committee (EA2/097/21).

Survey results were evaluated with descriptive statistics. Correlations between two factors were examined using the Chi-squared test. *P* values < 0.05 were considered statistically significant. All reported *p* values are two-sided.

## Results

In total, 116 physicians completed the survey (Table 1). All respondents answered all required questions. Most participants (57%) were 41–60 years old. The majority (95%) were gynecologists and worked at academic (66%) or university (25%) hospitals. Most respondents (78%) worked in a higher position, such as senior physician or head of department, and in a certified breast cancer center (93%). Forty-three percent of participating physicians stated that one or more colleagues at their department were breast ultrasound specialists of at least DEGUM II level (DEGUM = German Society for Ultrasound in Medicine).

**Table 1:** Sociodemographic data of respondents

Question	*n* (%)
Sex	
Female	73 (63%)
Male	42 (36%)
Diverse	1 (1%)
Age	
< 30 years old	2 (2%)
30–40 years old	29 (25%)
41–50 years old	33 (28%)
51–60 years old	34 (29%)
> 60 years old	18 (16%)
Specialty	
Gynecology	109 (95%)
Radiology	6 (5%)
Department type	
University hospital	29 (25%)
Academic hospital	76 (66%)
Hospital without academic affiliation	10 (9%)
Practice/outpatient clinic	9 (8%)
Mammography screening	2 (2%)
Current position	
Resident	9 (8%)
Specialist	16 (14%)
Senior Physician	57 (49%)
Head of Department	34 (29%)
Is your department part of a certified breast cancer center?	
Yes	108 (93%)
No	8 (7%)
Number of breast cancer cases treated at the department per year	
< 100	5 (4%)
100–200	52 (45%)
201–300	26 (22%)
301–400	16 (14%)
> 400	16 (14%)
Highest DEGUM^a^ breast ultrasound qualification available at the department	
None	36 (31%)
DEGUM I	30 (26%)
DEGUM II	42 (37%)
DEGUM III	7 (6%)

^a^German Society of Ultrasound in Medicine (www.degum.de)

### Axillary management in cN0 patients

In patients with clinically negative node status scheduled to receive NACT, 89% of respondents recommended SLNB after NACT (Supplementary Table 2). Four respondents stated that they perform SLNB both before and after NACT, depending on the individual case. They were asked to describe which factors this decision might depend on, and named the following:

- small HER2-positive tumors when pre-NACT SLNB may impact therapy choice,
- dependent on ultrasound findings,
- in case of a planned mastectomy to assess the indication for radiation therapy (in this case, SLNB may impact choice of reconstructive technique),
- age, prognosis.

In case of a micrometastasis in a sentinel node after NACT, most respondents (44%) recommended no further axillary therapy, while 31% proposed completion ALND and 25% irradiation of the axilla.

### Axillary management in cN + patients

In case of suspicious axillary nodes at the time of diagnosis, 64% of respondents recommended a minimally invasive biopsy to all patients, confirming the nodal status, while 34% advised it to selected patients only, and did not offer minimally invasive biopsy in case of unequivocally positive node status upon imaging (*n* = 19), in cases with high tumor load in the axilla including level I to III (*n* = 27), in cases with at least 2 (*n* = 4) or at least 4 (*n* = 13) suspicious nodes (Table 2). Other reasons for the omission of a confirmative biopsy were nodes located in a direct proximity of blood vessels, leading to an increased complication risk (*n* = 3) and high patient age (*n* = 2). The vast majority performed core needle biopsy instead of fine needle aspiration. TAD was recommended to most patients converting from a clinically positive to a clinically negative node status through NACT (cN +  → ycN0) by 78% of respondents, followed by ALND (16%) and SLNB (5%). None of the respondents chose targeted lymph node biopsy as the technique of choice in this setting.

**Table 2:** Current approach to axillary treatment in cN + patients in the neoadjuvant setting

Question	*n* (%)
Do you recommend cN + patients a minimally invasive confirmation of lymph node status?	
No	2 (2%)
Yes, always	74 (64%)
Yes, but not in all patients	40 (34%)
Which technique of minimally invasive biopsy do you usually perform?	
Core biopsy	106 (91%)
Fine needle aspiration	5 (4%)
I do not perform minimally invasive biopsies	5 (4%)
Which axillary staging technique do you recommend for most of your cN + patients converting to ycN0 status?	
Axillary lymph node dissection	19 (16%)
Targeted axillary dissection (TAD)	91 (78%)
Sentinel lymph node biopsy	6 (5%)
Targeted lymph node biopsy	0 (0%)

Nearly all respondents were familiar with TAD, and 82% stated that the technique was offered at their department (Table 3). In 47% of cases, considerable experience with TAD has been gathered so far (at least 30 procedures performed), and 70% of respondents reported that their department takes part in or plans to join the international AXSANA study (http://axsana.eubreast.com) [8]. While the majority of respondents recommended TAD to all cN + patients converting to ycN0, 43% offered the technique to selected patients only. This group was asked which factors influenced their decision whether to offer TAD or not. The free-text answers were (multiple answers were allowed):

- number of suspicious nodes (85%); 18 respondents reported that they offer TAD to patients with:
  o max. 1 suspicious node: 2 (11%),
  p max. 2 suspicious nodes: 9 (50%),
  q max. 3 suspicious nodes: 5 (28%),
  r max. 4 suspicious nodes: 2 (11%),
- inflammatory breast cancer (5%),
- good response to NACT in the breast (2%),
- tumor biology (2%),
- age (2%),
- depending on the surgeon (7%).

**Table 3:** Current approach to targeted axillary dissection

Question	*n* (%)
How much experience does your department have with targeted axillary dissection?	
I have never heard of this technique	1 (1%)
I have heard of this technique, but it is not used in my department	20 (17%)
TAD is used in my department	94 (82%)
How many TAD procedures have been performed in your department so far?	
< 30	50 (53%)
≥ 30	44 (47%)
Do you offer TAD to all or only selected cN + → ycN0 patients?	
All	54 (57%)
Selected	41 (43%)
Do you perform frozen section of target and sentinel lymph node(s)?	
Yes	44 (45%)
No	54 (55%)
Does your department participate in the AXSANA EUBREAST-3 study?	
Yes, already registered	64 (56%)
Yes, study participation planned	16 (14%)
No	35 (30%)

*TAD* targeted axillary dissection

### Marking techniques

While the marking of axillary lymph node(s) in cN + patients before NACT was supported by the majority of respondents (Table 4), it was used in selected patients only by 37%. The main factor influencing this decision was the number of initially suspicious lymph nodes. The marking procedure was undertaken during minimally invasive biopsy, i.e., as one procedure, by 53%, while 25% delayed the procedure until the histological/pathological report was available. The remaining 22% of respondents reported that the time point of lymph node marking may depend on:

- the probability of node metastasis,
- the indication for NACT,
- the indication for TAD,
- patient preference.

**Table 4:** Current approach to lymph node marking

Question	*n* (%)
Do you recommend lymph node marking to your cN + patients before NACT?	
No	11 (10%)
Yes, to all patients	61 (53%)
Yes, to selected patients	43 (37%)
Which marking technique do you currently use?	
Ink	4 (4%)
Magnetic seeds (e.g., MagSeed)	5 (5%)
Radioactive seeds	4 (4%)
RFID Tags (Radiofrequency marker, e.g., LOCalizer)	1 (1%)
Radar-based markers (e.g., SaviScout)	1 (1%)
Clips/coils	87 (85%)
When do you mark lymph nodes?	
At time of minimally invasive biopsy	54 (53%)
After the histological/cytological report	26 (25%)
Both at time of minimally invasive biopsy and after the histological/cytological report	22 (22%)
How many lymph nodes do you mark, if more then one node is suspicious?	
One node	43 (42%)
Two nodes	34 (33%)
Three or more nodes	3 (3%)
Depends on other factors	22 (22%)

*NACT* neoadjuvant chemotherapy

In patients presenting with more than one suspicious node at time of diagnosis, 42% of respondents recommended marking of only one node, while 33% stated that they mark two nodes in such cases. A fifth (22%) answered that the number of marked nodes may vary and that factors influencing the number of marked nodes were, e.g., the radiologist’s assessment or the number of biopsied nodes.

The most common marking technique was a clip/coil, used by 85% of respondents. Probe-guided detection techniques such as magnetic, radar reflecting, or radiofrequency markers were used less commonly (Table 4). Among clips/coils, different shapes and types are used:

- Tumark Vision: 38% (*n* = 23),
- HydroMark: 29% (*n* = 17),
- O-TWIST: 27% (*n* = 15),
- Müller-Schimpfle-Coil: 4% (*n* = 2),
- KliniMark: 2% (*n* = 1),
- Tumark Professional: 2% (*n* = 1).

Among respondents using clips/coils for lymph node marking, 86% performed preoperative wire localization and 42% intraoperative ultrasound to identify target lymph nodes. In case a clip/coil cannot be visualized on ultrasound, 54% recommended additional imaging, usually mammography or computed tomography, while the remaining 46% did not.

### Detection rates of different markers

**Fig. 1:**
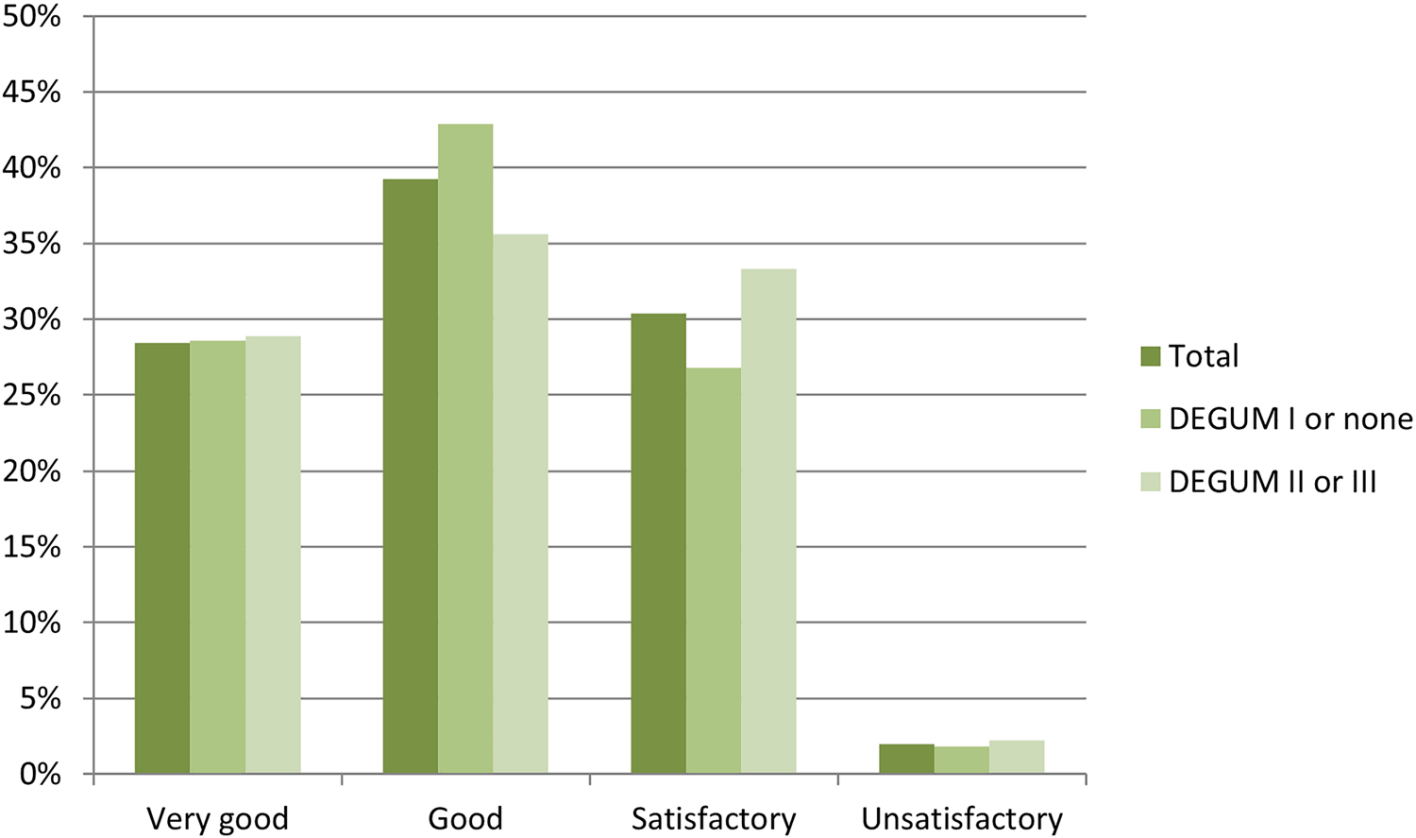
Estimation of the detection rate of lymph node marker used by respondents, depending on the highest breast ultrasound qualification at the respondent’s department

While 67% of respondents estimated the detection rate of their marker to be very good or good, 30% reported it was satisfactory and 2% that it was unsatisfactory (Supplementary Table 3). This was independent of the highest breast ultrasound qualification at the respondent’s department (Fig. 1). In case of ink, magnetic seeds, radar reflecting markers and RFID tags, all survey participants described the detection rate as good or very good. In contrast, only 65% of respondents using clips/coils reported a good or very good detection rate. Detection rates of different clip/coil types are presented in Supplementary Table 4. Due to small absolute numbers, a reliable comparison of detection rates using different markers was not possible.

### “Lost marker”

Thirty-nine out of 102 (38%) respondents reported that it had occurred at their department at least once that the retrieval of a marker placed before NACT could not be confirmed at surgery. Among these, 15 participants stated that no postoperative imaging was performed so that it remained unclear whether the marker was still residing in the patient or not. The remaining 24 respondents reported that in some cases, postoperative imaging confirmed marker removal at surgery, while in others the marker was still in situ. Different imaging modalities such as mammography / X-ray, (low dose) CT and ultrasound were used to search for the lost marker. When asked about the clinical consequences of a residual marker, 13 survey respondents answered that a second surgery was discussed individually with the patient.

### MRI artifacts

Respondents using magnetic, radar reflecting, or radiofrequency markers were asked two additional questions about MRI after marker placement. One respondent using RFID tags and two using magnetic markers reported MRI artifacts, others did not perform MRI after marker placement. All three reported that the evaluation of MRI was “somewhat limited” due to artifacts.

### Which factors influence attitudes of surgeons and radiologists towards axillary management?

High-volume departments were significantly more likely to employ at least one physician with an ultrasound qualification of DEGUM II or III (*p* < 0.001). DEGUM II/III ultrasound specialists were part of the team in 72% of departments treating over 300 cases per year. In contrast, only 34% of centers with ≤ 200 breast cancer cases per year had DEGUM II/III qualified staff. There was no correlation between highest DEGUM qualification in the department and attitudes of respondents towards axillary management in cN0 and cN + patients. However, departments with DEGUM II/III qualification were significantly more likely to have experience with TAD compared to those with highest qualification DEGUM I or none (92 vs. 74% respectively, *p* = 0.018). In departments performing TAD, the number of procedures conducted so far did not correlate with the highest DEGUM qualification.

**Fig. 2:**
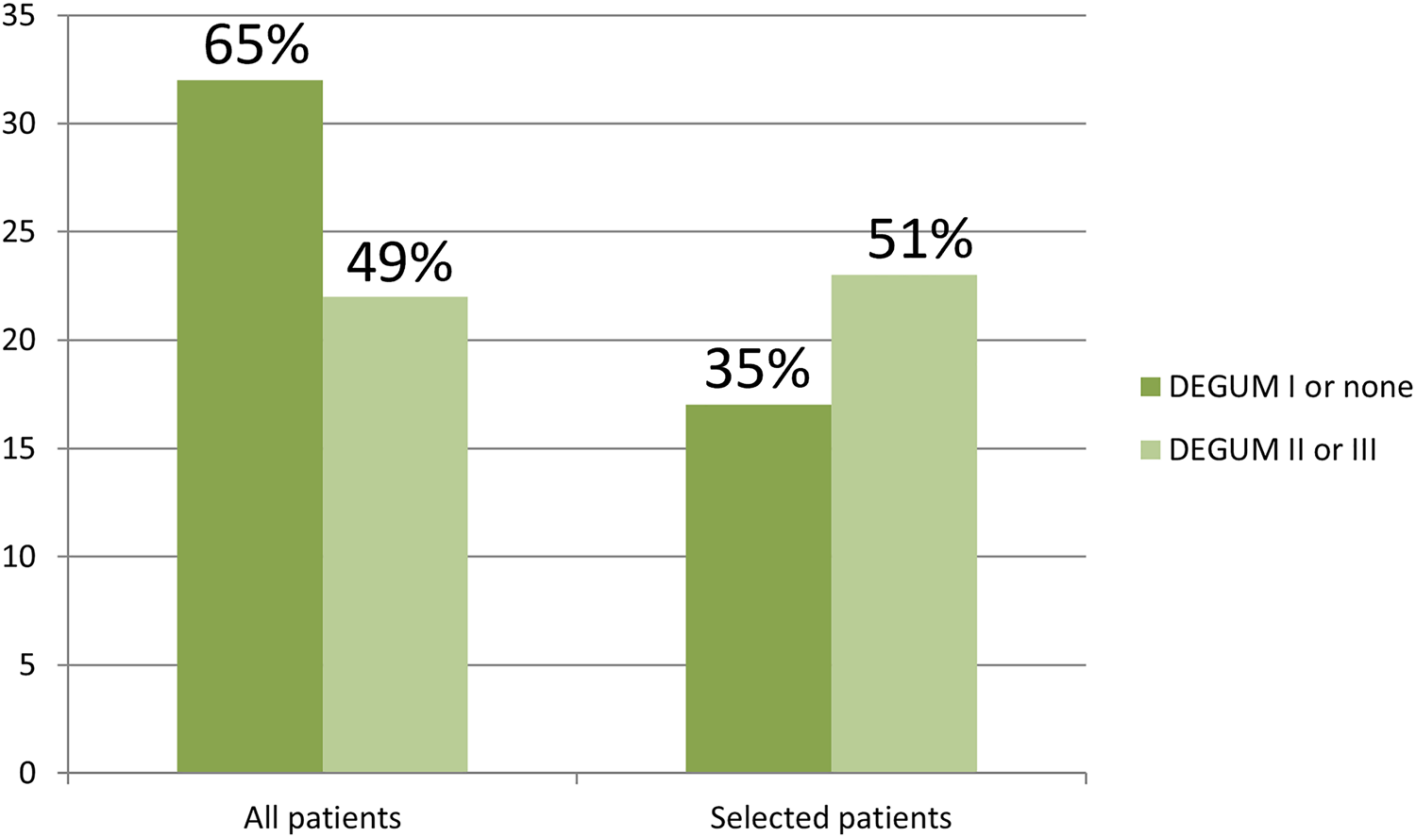
Distribution of answers to the question: “Do you offer TAD to all or only selected cN +  → ycN0 patients?” in relation to the highest ultrasound qualification available at the respondent’s department

Interestingly, while 51% of respondents working in DEGUM II/III departments reported to offer TAD to selected but not all cN +  → ycN0 patients, 65% of participants from departments without DEGUM II/III qualification offered TAD to all patients (Fig. 2). This difference was not significant (*p* = 0.108). Respondents from larger departments were significantly more likely to offer TAD to selected patients only than those from smaller centers (39.3% in departments treating over 300 vs. 64.2% in ≤ 300 breast cancer cases per year, *p* = 0.026).

Respondents’ age, position and sex did not influence their attitudes towards axillary management. Answers did not correlate with the department type (university/academic vs. other).

## Discussion

This is the first nationwide survey on physicians’ attitudes towards axillary management in the neoadjuvant setting in Germany. While there was consensus on many currently discussed topics such as the correct timepoint of SLNB in cN0 patients or the necessity to perform minimally invasive node biopsy in case of suspicious nodes, answers varied strongly with regard to specific clinical scenarios both in the cN0 and cN + setting.

Interestingly, a minority recommended completion ALND for initially cN0 patients with micrometastasis in the SLNB after NACT. This seems surprising since the AGO Breast Committee clearly recommends an ALND in case of ypN1mi status (available at: www.ago-online.de; Supplementary Fig. 1) [9]. The reason for this recommendation is the high rate of positive non-SLNs (64%) shown in previous studies [10]. The high number of respondents who omit any axillary intervention in this situation may be due to a common misunderstanding resulting from divergent recommendations in the primary surgery versus the neoadjuvant setting. While current guidelines uniformly recommend no further axillary surgery in patients with micrometastatic sentinel lymph node(s) receiving primary surgery [1, 2, 11], ALND is supported in low-volume residual axillary disease after NACT due to the proposed difference in biological relevance of resistant versus upfront disease [10].

In initially node-positive patients, the present survey revealed a heterogenous approach to axillary treatment. This may be due to heterogenous guidelines recommendations on the national and international level [5]. Indeed, recommendations commonly followed in Germany differ on the optimal surgical technique for patients converting from initially clinically positive to negative lymph node status. While the AGO Breast Committee recommends both TAD and ALND for this patient group [1, 12, 13], the S3 guideline recommends ALND for all cN + patients, irrespective of the clinical response of axillary nodes [7]. Most respondents recommended TAD to the majority of their cN +  → ycN0 patients, followed by ALND and SLNB. None recommended targeted lymph node biopsy, i.e., removal of marked target lymph node without SLNB, a technique first introduced as “MARI procedure” in the Netherlands, and not endorsed by guidelines [14].

While the majority of respondents from departments performing TAD viewed the procedure as technique of choice for all patients converting from cN + to ycN0 status, treatment choices correlated with department size and breast ultrasound experience. Thus, participants from high-volume centers were significantly more likely to recommend TAD to selected patients only. Similarly, numerically more respondents employed at centers with at least one DEGUM II/III breast ultrasound specialist recommended TAD to selected patients. The most frequently named factor influencing the decision for or against TAD was the number of suspicious nodes at time of diagnosis and the maximum number varied between one and four. These discrepancies may reflect the recent changes in the AGO Breast Committee guidelines. In March 2022, the AGO Breast Committee upheld the “ + ” recommendation for TAD in patients with initially 1–3 suspicious nodes but lowered the recommendation grade in patients with ≥ 4 suspicious nodes to “ ± “ (Supplementary Fig. 2). While data on the false-negative rate of TAD in relation to the number of initially involved nodes are lacking, the heterogenous response of lymph nodes to NACT is a well-known fact, and it is thus rational to hypothesize that the higher the number of suspicious nodes at presentation, the more probable it is for the TAD to miss a residual nodal metastasis [15, 16].

Various nodal marking techniques were used by the respondents. Views on the optimal number of nodes to be marked in patients presenting with more than one suspicious node varied widely. This reflects the current position of the AGO Breast Committee, stating that there is not enough evidence to recommend marking of one or more nodes in this setting (Supplementary Fig. 2). While the majority of respondents estimated the detection rate of the marker used at their department as very good or good, others deemed it only satisfactory or unsatisfactory. Since most centers use clips/coils to mark the target node, this is in line with findings from the SENTA trial, the largest study to date on clip-based TAD, showing relatively low detection rates of target lymph nodes (329 out of 423, 78%) [17]. In fact, the true detection rate was actually slightly lower, since targeted node excision was not attempted in further 34 patients because the clip was not visible upon ultrasound. In this context, the “lost marker” situation evoked different individual approaches reaching from further imaging or a new surgical intervention to no intervention at all. In the CLIP study, the clip could neither be detected by intraoperative radiograph nor by pathological evaluation of the excised axillary tissue in 33% of patients, and the lost clip was postoperatively detected in the patient’s axilla in only 20% of these cases. In the remaining 80% of patients, neither mammography nor CT-scan of the chest demonstrated clips in situ, suggesting that they might have been unnoticeably removed from the surgical cavity by swabs or suction [18]. The AGO Breast Committee recommends an ALND in patients in whom the marker cannot be identified but discourages from further invasive procedures to retrieve a lost marker (Supplementary Fig. 2).

## Conclusion

The present study reports a heterogenous approach towards axillary diagnostics and treatment in Germany. The vast majority of respondents follows current guidelines on issues such as the optimal timepoint of sentinel node excision in the neoadjuvant setting and the necessity to perform minimally invasive lymph node biopsy in case of suspicious nodes. However, clinical decisions and treatment guidelines differ strongly in case of patients with micrometastasis in the sentinel node after NACT. Further, a strong heterogeneity with regard to treatment of cN +  → ycN0 disease was observed, with different marking and localization techniques as well as surgical strategies of choice. Since only two-thirds of respondents described the detection rate of the marker used at their department as very good or good, future studies should focus on the comparative evaluation of different marking techniques.

## Supplementary Information

Below is the link to the electronic supplementary material.

Supplementary file1 (PNG 121 KB)

Supplementary file2 (PNG 95 KB)

Supplementary file3 (DOCX 26 KB)
