# Supplementary figures and images for: Current trends in diagnostic and therapeutic management of the axilla in breast cancer patients receiving neoadjuvant therapy: results of the German-wide NOGGO MONITOR 24 survey
Source: Arch Gynecol Obstet. 2022 Oct 10;307(5):1547–56. doi: 10.1007/s00404-022-06804-w (PMC10110637; doi:10.1007/s00404-022-06804-w)

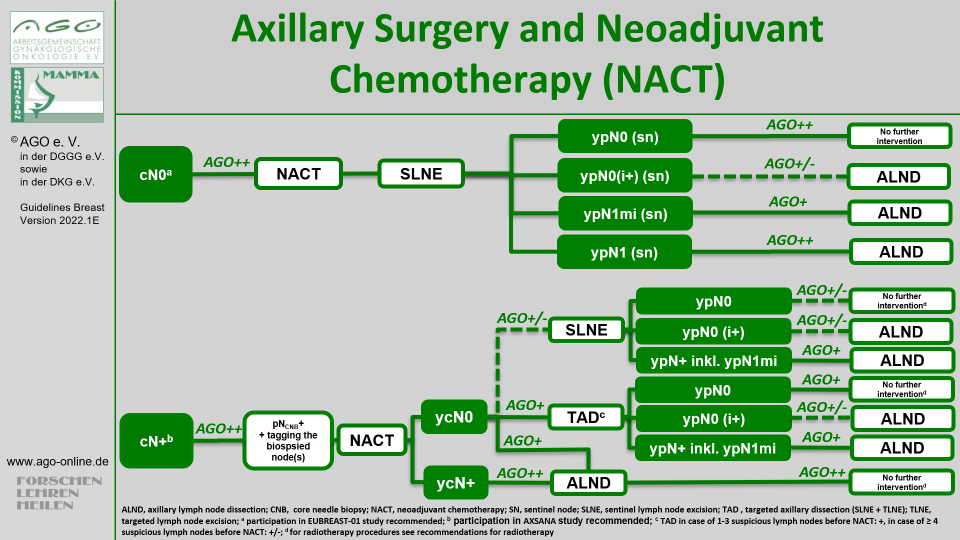

Supplement: Supplementary file 1 — Supplementary file1 (PNG 121 KB) [file 404_2022_6804_MOESM1_ESM.png]

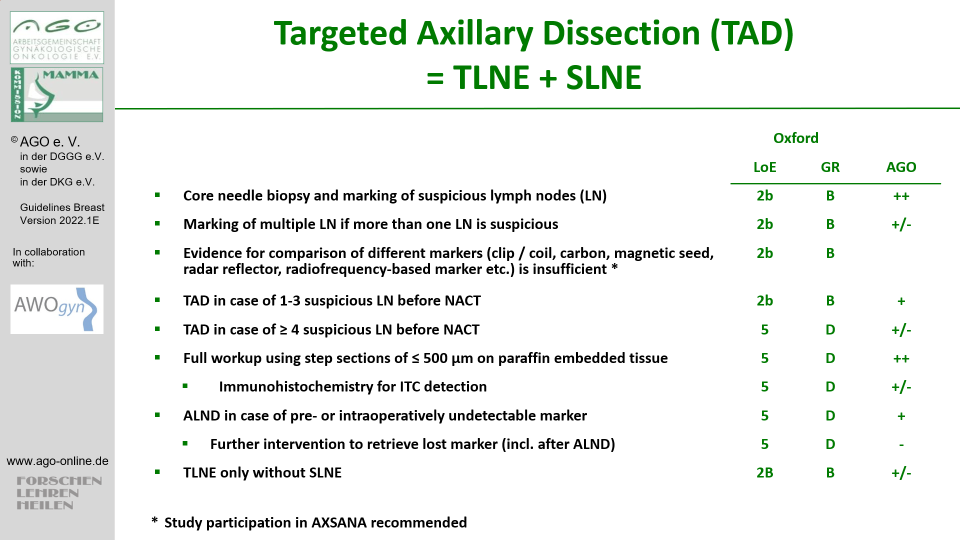

Supplement: Supplementary file 2 — Supplementary file2 (PNG 95 KB) [file 404_2022_6804_MOESM2_ESM.png]
