# Supplementary material for: Current trends in diagnostic and therapeutic management of the axilla in breast cancer patients receiving neoadjuvant therapy: results of the German-wide NOGGO MONITOR 24 survey
Source: Arch Gynecol Obstet. 2022 Oct 10;307(5):1547–56. doi: 10.1007/s00404-022-06804-w (PMC10110637; doi:10.1007/s00404-022-06804-w)
Supplement: Supplementary file 3 — Supplementary file3 (DOCX 26 KB) [file 404_2022_6804_MOESM3_ESM.docx]

**Supplementary material**

**Supplementary Table 1.** NOGGO MONITOR 24 Questionnaire.

|  | **English translation** | **German original** |
| --- | --- | --- |
| 1 | Sex:  - Female  - Male  - Diverse | Was ist Ihr Geschlecht?  - Weiblich  - Männlich  - Divers |
| 2 | How old are you?  - < 30 years old  - 30-40 years old  - 41-50 years old  - 51-60 years old  - > 60 years old | Wie alt sind Sie?  - < 30 Jahre  - 30-40 Jahre  - 41-50 Jahre  - 51-60 Jahre  - > 60 Jahre |
| 3 | In which state do you live in Germany? | In welchem Bundesland arbeiten Sie? |
| 4 | Which is your specialty?  - Gynecology  - Radiology  - Other (please name your specialty): | Welcher Fachrichtung gehören Sie an?  - Gynäkologie  - Radiologie  - Sonstiges (bitte angeben): |
| 5 | Which department are you working in? (Multiple choice possible)  - University hospital  - Academic hospital  - Hospital without academic affiliation  - Practice / outpatient clinic  - Mammography screening | In welcher Einrichtung arbeiten Sie? (Mehrfachauswahl möglich)  - Universitätsklinik  - Akademisches Lehrkrankenhaus  - Krankenhaus ohne Lehrauftrag  - Praxis / MVZ  - Mammographie-Screening |
| 6 | Which is position?  - Specialty trainee / Resident  - Specialist  - (Chief) Senior Physician  - Head of Department | In welcher Funktion sind Sie tätig?  - Assistenzärztin / Assistenzarzt  - Fachärztin / Facharzt  - (Leitende) Oberärztin / (Leitender) Oberarzt  - Chefärztin / Chefarzt |
| 7 | Is your department part of a certified breast cancer center?  - Yes  - No | Ist Ihre Einrichtung Teil eines zertifizierten Brustkrebszentrums?  - Ja  - Nein |
| 8 | How many breast cancer patients are treated at your department per year?  - < 100  - 100-200  - 201-300  - 301-400  - > 400  - Not applicable, my department conducts only diagnostics | Wie viele Patientinnen mit Mammakarzinom werden in Ihrer Einrichtung pro Jahr behandelt?  - < 100  - 100-200  - 201-300  - 301-400  - > 400  - Nichtzutreffend, meine Einrichtung führt ausschließlich Diagnostik durch |
| 9 | Do you or one of your co-workers have a DEGUM (German Society of Breast Ultrasound in Medicine) breast ultrasound certification? If yes, which is the highest DEGUM certification level in your department?  - No  - Yes, DEGUM I  - Yes, DEGUM II  - Yes, DEGUM III | Sind Sie bzw. Ihre Mitarbeiter als DEGUM-Stufeninhaber Mammasonographie zertifiziert? Wenn ja, geben Sie die höchste DEGUM-Stufe an, die in Ihrer Abteilung vorhanden ist.  - Nein  - Ja, DEGUM-Stufe I  - Ja, DEGUM-Stufe II  - Ja, DEGUM-Stufe III |
| 10 | When do you perform SLNB in cN0 patients and recommendation for NACT?  - Always before NACT  - Always after NACT  - Both before and after NACT, depending on the individual case:  Which factors influence your decision? _______________________ | Wann führen Sie die Sentinel-Lymphonodektomie bei cN0-Patientinnen und Indikation zur neoadjuvanten Chemotherapie (NACT) durch?  - Immer vor der NACT  - Immer nach der NACT  - Sowohl vor als auch nach der NACT:  Von welchen Faktoren machen Sie es abhängig? ___________ |
| 11 | Which therapy do you recommend patients with micrometastasis in the sentinel node after NACT?  No further axillary therapy  Axillary radiation  Axillary lymph node dissection (ALND) | Welches Vorgehen empfehlen Sie jenen initial cN0-Patientinnen, bei denen die Sentinel-Lymphonodektomie nach der NACT eine Mikrometastasierung ergab (ypN1mi)?  - Keine weitere axilläre Therapie  - Radiatio der Axilla  - Axilladissektion (ALND) |
| 12 | Do you recommend cN+ patients a minimally invasive confirmation of lymph node status?  - No  - Yes, always  - Yes, but not in all patients | Empfehlen Sie den cN+ Patientinnen eine minimal-invasive Sicherung der Lymphknotenmetastasierung?  - Nein  - Ja, immer  - Ja, aber nicht bei allen Patientinnen |
| 13 | In which setting would you NOT perform or recommend a minimally invasive node biopsy in a cN+ patient? (multiple choice)  - Unequivocally positive node status upon imaging  - High axillary tumor load (e.g., level I to III)  - At least 2 highly suspicious nodes  - At least 4 highly suspicious nodes  - Other, please specify: ______ | In welcher Situation würden Sie bei cN+ Status KEINE minimal-invasive Sicherung der Lymphknoten durchführen bzw. empfehlen? (Mehrfachantwort möglich)  - Bildgebend eindeutiger Nodalbefall  - Sehr ausgeprägter Nodalbefall (z.B. Level I-III)  - Mindestens 2 hochsuspekte Lymphknoten  - Mindestens 4 hochsuspekte Lymphknoten  - Andere, welche: ___________ |
| 14 | Which technique of minimally invasive biopsy do you usually perform?  - Core biopsy  - Fine needle aspiration  - I do not perform minimally invasive biopsies | Welche Technik der minimal-invasiven Sicherung der Lymphknoten führen Sie am häufigsten durch?  - Stanzbiopsie  - Feinnadelaspiration  - Ich führe keine minimal-invasiven Biopsien durch |
| 15 | Which axillary staging technique do you recommend for most of your cN+ patients converting to ycN0 status?  - Axillary lymph node dissection (ALND)  - Targeted axillary dissection (TAD)  - Sentinel lymph node biopsy  - Targeted lymph node biopsy | Welche Form der Axilla-Operation empfehlen Sie den meisten initial cN+ Patientinnen, die unter der neoadjuvanten Therapie zum ycN0-Status konvertieren?  - Axilladissektion (ALND)  - Targeted axillary dissection (TAD = Entfernung des Target-Lymphknotens und des Sentinel-Lymphknotens)  - Nur Sentinel-Lymphonodektomie  - Nur Entfernung des Target-Lymphknotens |
| 16 | How much experience does your department have with targeted axillary dissection?  - I have never heard of this technique  - I have heard of this technique, but it is not used in my department  - TAD is used in my department | Wieviel Erfahrung gibt es in Ihrer Einrichtung mit der Targeted axillary dissection?  - Ich habe von dieser Technik noch nie gehört.  - Ich habe von dieser Technik gehört, sie wird aber in meiner Einrichtung nicht verwendet.  - In meiner Einrichtung wird diese Technik verwendet. |
| 17 | How many TAD procedures have been performed in your department so far?  - < 30  - ≥ 30 | Wie viele TAD-Operationen wurden in Ihrer Einrichtung bereits durchgeführt?  - < 30  - ≥ 30 |
| 18 | Do you offer TAD to all or only selected cN+ → ycN0 patients?  - All  - Selected:  Which factors influence your decision? __________________ | Bieten Sie die TAD allen oder nur ausgewählten cN+ → ycN0 Patientinnen an?  - Allen  - Nur ausgewählten:  Von welchen Faktoren hängt Ihre Entscheidung ab? ______________ |
| 19 | Do you recommend lymph node marking to your cN+ patients before NACT?  - No  - Yes, to all patients  - Yes, to selected patients:  Which factors influence your decision? __________________ | Empfehlen Sie Ihren cN+ Patientinnen eine Lymphknoten-Markierung vor Beginn der neoadjuvanten Chemotherapie?  - Nein  - Ja, allen Patientinnen  - Ja, ausgewählten Patientinnen:  Von welchen Faktoren hängt Ihre Entscheidung ab? ___________ |
| 20 | Does your department participate in the AXSANA EUBREAST-3 study?  - Yes, already registered  - Yes, study participation planned  - No | Nehmen Sie an der AXSANA-Studie teil?  - Ja, bereits angemeldet  - Ja, geplant  - Nein |
| 21 | Which marking technique do you currently use?  - Ink  - Magnetic seeds (e.g., MagSeed)  - Radioactive seeds  - RFID Tags (Radiofrequency marker, e.g., LOCalizer)  - Radar-based markers (e.g., SaviScout)  - Clips/Coils: which type exactly? _______ | Welche Lymphknoten-Markierungstechnik verwenden Sie derzeit?  - Farbstoff  - Magnetische Seeds (z.B. MagSeed)  - Radioaktive Seeds  - RFID Tags (Radiofrequenz-Marker, z.B. LOCalizer)  - Radar-basierte Marker (z.B. Savi Scout)  - Clip/Coil: welche genau? ___________ |
| 22 | When do you mark lymph nodes?  - At time of minimally invasive biopsy  - After the histological/cytological report  - Both at time of minimally invasive biopsy and after the histological/cytological report:  Which factors influence your decision? __________________ | Wann markieren Sie den/die Lymphknoten?  - Im Rahmen der histologischen Sicherung  - Nach Erhalt des zytologischen/histologischen Befundes  - Unterschiedlich, sowohl im Rahmen der Biopsie als auch nach Erhalt des Befundes:  Von welchen Faktoren hängt Ihre Entscheidung ab? ___________ |
| 23 | How many lymph nodes do you mark, if more then one node is suspicious?  - One node  - Two nodes  - Three or more nodes  - Depends on other factors:  Which factors influence your decision? __________________ | Wie viele Lymphknoten markieren Sie, wenn mehrere Lymphknoten suspekt sind?  - Einen Lymphknoten  - Zwei Lymphknoten  - Drei oder mehr Lymphknoten  - Unterschiedlich:  Von welchen Faktoren hängt Ihre Entscheidung ab? ___________ |
| 24 | How good is the detection rate of the marker you use?  - Very good  - Good  - Satisfactory  - Unsatisfactory | Wie schätzen Sie die Detektionsrate des von Ihnen verwendeten Markers ein?  - Sehr gut  - Gut  - Befriedigend  - Nicht zufriedenstellend |
| 25 | Has it occurred in your department that a marker has not been retrieved during TAD or ALND (“Lost marker”)?  - No  - Yes, but a postoperative imaging was not performed, so it remains unclear whether the marker is still in the patient or not  - Yes, and postoperative imaging was performed: Which imaging and what was the result? __________ | Ist es in Ihrer Einrichtung schon mal vorgekommen, dass der Marker während der TAD bzw. ALND nicht entfernt wurde („Lost Marker“)?  - Nein  - Ja, es wurde aber keine postoperative Bildgebung veranlasst, sodass unklar bleibt, ob sich der Marker noch im Körper der Patientin befindet  - Ja und es wurde postoperativ weitere Bildgebung veranlasst:  Welche und was hat sie ergeben? ___________ |
| 26 | What did you recommend this patient?  - Another surgery/intervention to retrieve marker  - No further surgery/intervention  - Not applicable (no marker in patient’s body) | Was haben Sie der Patientin empfohlen?  - Eine erneute Operation/Intervention, um den Marker zu entfernen.  - Keine erneute Operation/Intervention.  - Trifft nicht zu (kein verbliebener Marker). |
| 27 | Do you perform frozen section of target and sentinel lymph node(s)?  - Yes  - No  - Not applicable (my department performs diagnostics only) | Veranlassen Sie während der TAD eine intraoperative Schnellschnittuntersuchung des Target- und Sentinel-Lymphknotens?  - Ja  - Nein  - Trifft nicht zu (in meiner Einrichtung wird ausschließlich Diagnostik durchgeführt) |
| To be answered only by participants using clip/coils: | | |
| 28 | How do you localize target lymph nodes pre- and intraoperatively? (multiple choice)  - Preoperative wire localization  - Intraoperative ultrasound  - No specific localization  - Other, which: ________ | Wie lokalisieren Sie den Target-Lymphknoten prä- bzw. intraoperativ? (Mehrfachantwort möglich)  - Präoperative Drahtlokalisation  - Intraoperative Sonographie  - Keine gezielte Lokalisation  - Andere, welche: ___________ |
| 29 | If the clip/coil cannot be visualized upon ultrasound, do you recommend any other form of localization?  - No  - Yes, which: ________ | Wenn Sie den Clip/Coil präoperativ sonographisch nicht darstellen können, veranlassen Sie eine andere Form der Lokalisation?  - Nein  - Ja, welche: ___________ |
| To be answered only by participants using magnetic, radar and radiofrequency markers: | | |
| 30 | If you performed breast MRI in one or more patients between node marking with a magnetic, radar or radiofrequency marker and surgery, did artifacts occur?  - Yes  - No  - I have not performed breast MRI after marking in any of my patients | Falls Sie bei einer oder mehreren Ihrer Patientinnen zwischen der Lymphknoten-Markierung mit magnetischem Seed, Radar-Marker oder Radiofrequenz-Marker und der Operation ein Mamma-MRT durchgeführt haben, kam es dabei zu MR-Artefakten?  - Ja  - Nein  - Ich habe bisher bei keiner meiner Patientinnen ein Mamma-MRT nach der Markierung durchgeführt |
| 31 | Was the assessment of breast MRI and evaluation of the clinical question limited due to artifacts?  - Yes, strongly limited  - Yes, somewhat limited  - No  - Not applicable (no breast MRI performed, or no artifacts produced) | War die Beurteilbarkeit des Mamma-MRT und die Beantwortung Ihrer Fragestellung durch die Artefakte eingeschränkt?  - Ja, stark eingeschränkt  - Ja, etwas eingeschränkt  - Nein  - Trifft nicht zu (kein Mamma-MRT oder keine Artefakte entstanden) |

**Supplementary Table 2.** Current approach to axillary treatment in cN0 patients in the neoadjuvant setting.

| **Question** | **n (%)** |
| --- | --- |
| When do you perform SLNB in cN0 patients and recommendation for NACT?  Always before NACT  Always after NACT  Both before and after NACT, depending on the individual case | 9 (8%)  103 (89%)  4 (3%) |
| Which therapy do you recommend patients with micrometastasis in the sentinel node after NACT?  No further axillary therapy  Axillary radiation  Axillary lymph node dissection (ALND) | 51 (44%)  29 (25%)  36 (31%) |

Abbreviations: NACT – neoadjuvant chemotherapy, SLNB – sentinel lymph node biopsy

**Supplementary** **Table 3.** Detection rate estimated by respondents, depending on the type of marker.

| **Type of marker** | **Detection rate** | **n (%)** |
| --- | --- | --- |
| Total | Very good  Good  Satisfactory  Unsatisfactory | 29 (28%)  40 (39%)  31 (30%)  2 (2%) |
| Ink | Very good  Good  Satisfactory  Unsatisfactory | 2 (50%)  2 (50%)  0  0 |
| Magnetic seeds (e.g., MagSeed) | Very good  Good  Satisfactory  Unsatisfactory | 3 (60%)  2 (40%)  0  0 |
| Radioactive seeds | Very good  Good  Satisfactory  Unsatisfactory | 1 (25%)  0  3 (75%)  0 |
| RFID Tags (Radiofrequency marker, e.g., LOCalizer) | Very good  Good  Satisfactory  Unsatisfactory | 0  1 (100%)  0  0 |
| Radar reflecting markers (e.g., SaviScout) | Very good  Good  Satisfactory  Unsatisfactory | 0  1 (100%)  0  0 |
| Clips/Coils | Very good  Good  Satisfactory  Unsatisfactory | 23 (26%)  34 (39%)  28 (32%)  2 (2%) |

Abbreviations: NACT – neoadjuvant chemotherapy

**Supplementary** **Table 4.** Detection rate estimated by respondents, depending on the exact type of clip/coil.

| **Type of marker** | **Detection rate** | **n (%)** |
| --- | --- | --- |
| HydroMark | Very good  Good  Satisfactory  Unsatisfactory | 5 (29%)  7 (41%)  5 (29%)  0 |
| O-TWIST | Very good  Good  Satisfactory  Unsatisfactory | 5 (33%)  6 (40%)  4 (27%)  0 |
| Tumark Vision | Very good  Good  Satisfactory  Unsatisfactory | 5 (22%)  10 (43%)  7 (30%)  1 (4%) |
| Müller-Schimpfle-Coil | Very good  Good  Satisfactory  Unsatisfactory | 2 (100%)  0  0  0 |
| KliniMark | Very good  Good  Satisfactory  Unsatisfactory | 0  0  1 (100%)  0 |
| Tumark Professional | Very good  Good  Satisfactory  Unsatisfactory | 0  1 (100%)  0  0 |
